# Are Ophiolitic Substrates Drivers for Reticulate Evolution in *Armeria* (Plumbaginaceae)?

**DOI:** 10.1002/ece3.71525

**Published:** 2025-06-12

**Authors:** Manuel Tiburtini, Salvatore Tomasello, Eleonora Manzo, Luca Sandroni, Thomas Abeli, Lorenzo Peruzzi

**Affiliations:** ^1^ PLANTSEED Lab, Department of Biology University of Pisa Pisa Italy; ^2^ Department of Systematics, Biodiversity and Evolution of Plants (With Herbarium), Albrecht‐von‐Haller Institute for Plant Sciences Georg‐August‐University of Göttingen Göttingen Germany; ^3^ Department of Earth and Environmental Science University of Pavia Pavia Italy

**Keywords:** *Armeria*, edaphism, endemism, phylogenomics, reticulate evolution, serpentine, speciation

## Abstract

Ultramafic substrates can play a role in fostering ecological adaptation and microevolutionary dynamics. The *Armeria denticulata* complex includes two flowering plant species ( *A. denticulata* and *A. saviana*): the former is a strict serpentinophyte endemic to Tuscany and western Liguria, while the latter grows on limestone/jasper in a small area of southern Tuscany. Intriguingly, northern Apennine populations of *A. arenaria* subsp. *praecox*, a subspecies otherwise endemic to the western Alps, grow on ophiolites. Finally, the central‐southern Italian endemic *A. gracilis* is instead linked to limestone. We aimed at understanding whether substrate specificity and/or hybridization promoted speciation in the *A. denticulata* complex, despite similar ecological conditions failing to cause speciation in the nearby *A. arenaria* . We used Genome skimming and Illumina sequencing to assemble plastomes (152 kb) and data from the nuclear genome (ribosomal DNA subunits and 36 single‐copy markers; 27 kb in total) used to infer phylogenies and evaluate different reticulate evolution scenarios by calculating gene tree probabilities under the Coalescent model. The phylogenomic analyses were complemented by morphometric data using a matrix of 134 individuals × 27 characters. Morphometric data were analyzed both by fitting a Gaussian Mixture Model to compute population‐wise Jensen–Shannon Distances and a Neighbor‐Net network, and by inferring a standard linear discriminant analysis. Both morphometric and phylogenomic results suggest *A. saviana* is a species of homoploid hybrid origin, involving *A. denticulata* s.str. (ovule donor) and *A. gracilis* (pollen donor). A single population of *A. denticulata* from inner Tuscany (Monte Ferrato) could have originated from an introgression/hybridization event between *A. denticulata* s.str. (pollen donor) and *A. arenaria* subsp. *praecox* (ovule donor). Accordingly, our results suggest that substrate specificity and hybridization/introgression prompted microevolutionary processes in the *Armeria denticulata* complex.

## Graphical abstract

The *Armeria denticulata* complex includes two species ( *A. denticulata* and *A. saviana*): The former is a strict serpentinophyte endemic to Tuscany and western Liguria, the latter grows on limestone/jasper in a small area of southern Tuscany. We aimed at understanding whether substrate specificity and/or hybridization promoted speciation in the *A. denticulata* complex, using phylogenomics and morphometric analyses. Both morphometric and phylogenomic results suggest that *A. saviana* is a species of homoploid hybrid origin, involving *A. denticulata* s.str. (ovule donor) and *A. gracilis* (pollen donor), whereas a single population of *A. denticulata* from inner Tuscany (Monte Ferrato) has originated from an introgression/hybridization event between *A. denticulata* s.str. (pollen donor) and *A. arenaria* subsp. praecox (ovule donor).

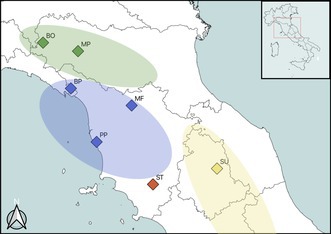

## 1 Introduction

Ophiolitic substrates, derived from ultramafic rocks such as peridotite and serpentinite, are unique for their element composition (Malpas 1992). Indeed, these soils are characterized by a high concentrations of heavy metals, such as nickel, chromium, and cobalt, and low levels of essential nutrients like calcium, potassium, phosphorus, and sulfur, and generally have a low calcium‐to‐magnesium (Ca:Mg) molar ratio (Marrs and Proctor 1976; Palm et al. 2012).

Interestingly, ophiolitic outcrops can appear as “islands” scattered through the landscape (Roberts and Proctor 2012). This situation is found, for instance, in Italy, Tuscany (Carmignani et al. 2013; Selvi 2007), California (Churchill and Hill 2000), New Caledonia (Isnard et al. 2016), and Cuba (González Gutiérrez et al. 2023), promoting landscape heterogeneity and consequently, biodiversity enrichment and endemism (Isnard et al. 2016).

Ophiolitic substrates have been identified as a significant selective factor in plant evolution (Kruckeberg 1951), promoting the evolution of ecotypes (Pavlova 2009) and speciation (Anacker 2014), making them good models for eco‐evo‐devo speciation (Harrison and Rajakaruna 2011). Indeed, the presence of toxic elements in ophiolitic substrates exerts strong selective pressures on plants, driving the evolution of unique physiological traits such as heavy metal tolerance (e.g., hyperaccumulation) and avoidance strategies (Cobbett 2003; Di Toppi et al. 2003; Rascio and Navari‐Izzo 2011). These pressures also influence morphological traits, often resulting in smaller, thicker leaves, reduced specific leaf area (SLA), and increased anthocyanin production to mitigate oxidative stress (Li and Ahammed 2023; Palm et al. 2012; Samojedny et al. 2022). Collectively, these adaptations fall under the concept of edaphism, which describes the role of soil properties in shaping plant morphology, distribution, and speciation (Rivas‐Goday 1969). Such edaphic factors are recognized as drivers of endemism and ecological diversification in multiple plant lineages (Mota et al. 2017; Nieto Feliner et al. 2002; Roberts and Proctor 2012).

The selective pressures are also evident in the formation of vegetation types exclusive to ophiolitic substrates (Cano et al. 2014; Roberts and Proctor 2012) that host a set of species strictly linked to such environments called serpentinophytes (Pichi‐Sermolli 1948).

Within this context, the genus *Armeria* Willd. (Plumbaginaceae) offers an excellent model for studying the evolutionary consequences of edaphic speciation. This genus includes 108 species (Malekmohammadi et al. 2024; POWO— 2017) that can grow in a broad set of different ecological niches. For example, *Armeria maritima* (Mill.) Willd. (Eisikowitch and Woodell 1975; Lefèbvre and Vekemans 1995; Weidema et al. 1996; Woodell and Dale 1993) can grow in both coastal areas and metal‐rich soil (Lefebvre 2021), such as mine dumps in Poland (Abratowska et al. 2012). *Armeria* species that grow on ophiolitic substrates seem prone to accumulate metals more in roots and leaves than in shoots (Tomović et al. 2018; Wierzbicka et al. 2023), and this is due to the limited access of such metals to the apoplastic diffusion in the shoots (Wierzbicka et al. 2023). Edaphism in *Armeria* is not rare. Indeed, many species in the Iberian Peninsula—a biodiversity hotspot for this genus—are strict serpentinophytes (e.g., *Armeria langei* Boiss. ex Lange subsp. *marizii* (Daveau) C.Aguiar, Sánchez‐Mata & Monteiro‐Henriques, *A. eriophylla* Willk., *A. colorata* Pau, etc.) (Nieto Feliner 1990).

Interestingly, homoploid hybridization and introgression played a crucial role in the evolution of this genus (Garcia et al. 2016; Nieto Feliner 1997; Nieto Feliner et al. 2017, 1996, 2019). This has led to considering some *Armeria* species as genetically “aggressive,” in capturing portions of the genome of other sympatric species through extensive introgression (Fuertes Aguilar et al. 1999), without losing reproductive fitness (Nieto Feliner et al. 1996). This process is also facilitated by a highly specialized self‐incompatibility system, which is regulated by pollen‐stigma dimorphism and chemical recognition (Baker 1966; Costa et al. 2017; Mattson 1983).

For example, *A. villosa* subsp. *carratracensis* (Bernis) Nieto Fel. is believed to have originated after a hybridization event between the serpentinophyte *A. colorata* , acting as the ovule donor, and the widespread *A. villosa* subsp. *longiaristata* (Boiss. & Reut.) Nieto Fel., growing on limestone, acting as the pollen donor (Nieto Feliner et al. 2002). Hybridization can facilitate the exchange of advantageous traits between species, fostering adaptation to stressful environments and enabling rapid evolutionary responses (Tauleigne‐Gomes and Lefèbvre 2005). This process is sometimes called “niche expansion” (Moore et al. 2021; Pfennig et al. 2016) and has been found to be a major driver of ecological adaptation in *Armeria* hybrids, as those between *A. pseudoarmeria* (Murray) Mansf. and *A. welwitschii* Boiss. in Portugal (Tauleigne‐Gomes and Lefèbvre 2005, 2008), or between *A. pungens* (Link) Hoffmanns. & Link and *A. macrophylla* Boiss. & Reut. in Spain (Nieto Feliner et al. 2019).

Chloroplasts are maternally inherited in *Plumbaginaceae* (Nieto Feliner et al. 2002), so that using cpDNA it is possible to reconstruct the phylogeny of the maternal line of descent, avoiding the confounding effects of introgression and hybridization on nrDNA (Vargas et al. 2017).

Regarding the Italian flora, 18 *Armeria* taxa occur, out of which 15 are endemic to the country (Bartolucci et al. 2024). Among them, only two species can grow on ophiolitic substrates. *Armeria arenaria* (Pers.) F.Dietr. subsp. *praecox* (Jord.) Kerguélen ex Greuter, Burdet & G.Long occurs throughout northern Italy and the French Alps (Tiburtini et al. 2022), and its southernmost distribution reaches the Northern Apennines, an area characterized by high soil heterogeneity (Carmignani et al. 2004, 2013), including ophiolitic substrates. This subspecies can be found on a variety of substrates, from granite, sandstones to serpentines, making it a “bodenvag” species *sensu* Kruckeberg (1951). In the Northern Apennines, *A. arenaria* subsp. *praecox* does not grow far from *A. denticulata* (Bertol.) DC. a narrow endemic taxon restricted to local serpentine outcrops (Selvi 2007). *Armeria denticulata* is commonly found in lowland serpentine outcrops throughout Tuscany, extending southwards to the Monte Amiata region, a dormant volcano (Marroni et al. 2015). There, it is geographically close to *A. saviana* Selvi (Selvi 2009), a species endemic to the surroundings of Monte Amiata, thriving mostly on sedimentary rocks such as limestone and jasper (Marroni et al. 2015) at altitudes higher than 900 m a.s.l. (Table 1). *Armeria denticulata* and *A. saviana* are closely related species, part of the so‐called *Armeria denticulata* complex (Pignatti et al. 2017), both endemic to Italy (Bartolucci et al. 2024). This complex shows unique morphological features that cannot be found in other Italian species, such as dentate leaves, broad and large triangular outer scales and small rosettes (Arrigoni 2015; Selvi 2009). *Armeria gracilis* Ten., another Italian endemic species, inhabits the Apennines more southwards, from the geographically close Umbria down to the Pollino mountain range in Calabria (Arrigoni 2015). This species typically grows in high montane calcareous pastures, up to 2500 m a.s.l.

**TABLE 1:** Information about species, locality, substrate, and voucher documentation of the studied *Armeria* populations. Sample size refers to the morphometric analyses. GenBank accession numbers for cp‐ and nrDNA sequences are provided, along with ENA sample number with embedded raw reads.

Population code	Species	Locality	Substrate	DNA voucher specimen	Sample size	cpDNA accession nr.	nrDNA accession nr.	ENA sample number
BO	*A. arenaria* subsp. *praecox*	Emilia‐Romagna, Bobbio (Piacenza)	Ophiolites	PI1788935–BO01	14	PV755955	PV755950	SAMEA115975757
MP	*A. arenaria* subsp. *praecox*	Emilia‐Romagna, Monte Prinzera, Fornovo del Taro (Piacenza)	Ophiolites	PI1788888–MP06	20	PV755956	PV755949	SAMEA115975758
MF	*A. denticulata*	Tuscany, Monte Ferrato (Prato)	Ophiolites	PI1780918–MF03	20	PV755958	PV755951	SAMEA115975759
BP	*A. denticulata*	Liguria, Brina di Ponzano, Sarzana (La Spezia)	Ophiolites	PI1780870–BP08	20	PV755957	PV755948	SAMEA115975760
PP	*A. denticulata*	Tuscany, Monte Pelato, Rosignano Marittimo (Livorno)	Ophiolites	PI1780958‐PP19	20	PV755959	PV755952	SAMEA115975761
ST	*A. saviana*	Tuscany, Pietra Sorbella, Arcidosso (Grosseto)	Limestone, jasper and other sedimentary rocks	PI1780894–ST06	20	PV755961	PV755954	SAMEA115975762
SU	*A. gracilis*	Umbria, Monte Subasio, Assisi (Perugia)	Limestone	PI2154269–SU04	20	PV755960	PV755953	SAMEA115975763

Given the allopatric distribution across different substrates and regions in the above‐mentioned group of *Armeria* taxa, we aim to answer the following two questions concerning the evolution of the *A. denticulata* complex: (1) does the *Armeria denticulata* complex form a monophyletic group, or is it phylogenetically intermingled with adjacent *Armeria* species occurring on distinct substrates? (2) If interspecific gene flow or introgression is detected, are there corresponding intermediate morphological features that support the occurrence of hybridization events? To address these questions, we used a combination of phylogenomic and morphometric analyses to shed light on the evolution and speciation patterns in the *Armeria* serpentinophyte taxa of central Italy.

## 2 Material and Methods

### 2.1 Plant Material

**FIGURE 1:**
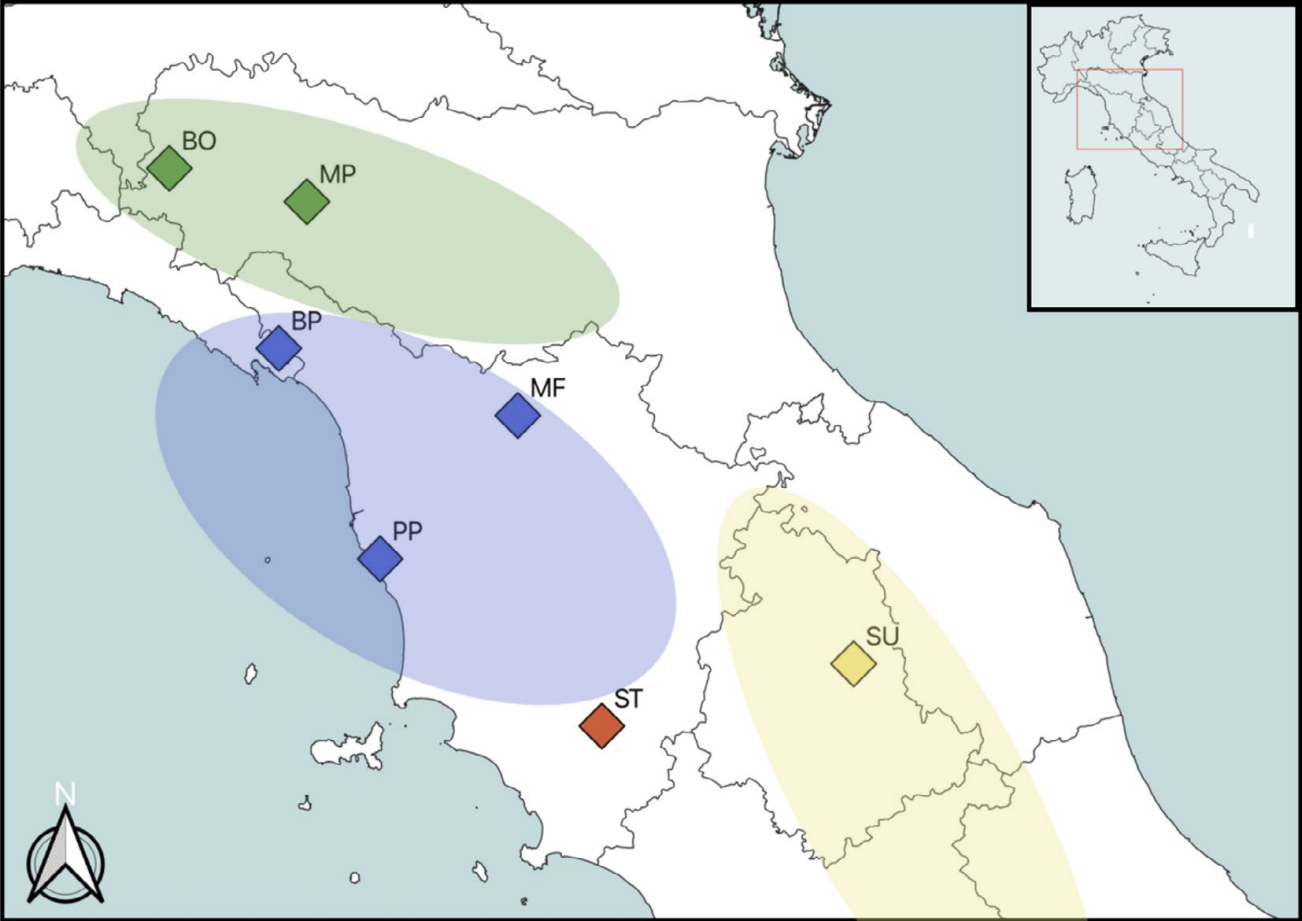
Map of studied populations. Green: *Armeria arenaria* ; blue: *A. denticulata* ; yellow: *A. gracilis* ; red: *A. saviana*. Ellipses indicate the approximate distribution of the four allopatric species in this portion of Italy.

We sampled seven populations from the *A. denticulata* complex and all neighboring congeneric species in Tuscany, Umbria, and Emilia‐Romagna (Figure 1). Leaves from one individual per population were sampled, placed in tea bags, and dried with silica gel for subsequent DNA extraction and sequencing. Digitized herbarium specimens of the sampled individuals are stored in the Herbarium Horti Botanici Pisani (PI) and are freely available for consultation at http://erbario.unipi.it/ (codes in Table 1). Around 20 individuals per population (134 in total) were utilized for morphometric analyses. All the studied species are diploid with 2*n* = 2*x* = 18 (Astuti et al. 2021; Brullo et al. 1995; Tiburtini et al. 2022; Tiburtini, Fruzzetti, et al. 2024).

### 2.2 Extraction and Illumina Library Preparation

We used 10–20 mg of dry leaves material for DNA extraction. For the scope, we used the Qiagen DNeasy Plant Mini Kit (Qiagen, Hilden, Germany), applying a few modifications as in Marinček et al. (2022). Accordingly, incubations in lysis and elution buffers were prolonged to 30′. Extracts were run in a 2% agarose gel to roughly estimate fragment lengths. Concentrations were measured using a Qubit 3 Fluorometer (Thermo Fisher Scientific) with the Qubit dsDNA HS Assay Kit (Thermo Fisher Scientific).

Library preparation for Illumina sequencing was carried out with the “NEBNext Ultra II FS DNA Library Prep Kit for Illumina” (New England BioLabs, Ipswich, Massachusetts, USA), setting the shearing step to 12′, in order to obtain DNA fragments ranging from 200 to 500 bp in length. After adapter ligation, samples underwent PCR amplification for 14 cycles and were barcoded with the “NEBNext Multiplex Oligos for Illumina (96 Unique Dual Index Primer Pairs)” (New England BioLabs). The amplified reactions were then purified with the HighPrep beads (MagBio Genomics, Gaithersburg, Maryland, USA) following the manufacturer's instructions. The quality of the libraries was checked on a QIAxcel advanced (Qiagen, Hilden, Germany) with the DNA High Resolution Kit 1200 (Qiagen), the “QX Size Marker 50bp‐800bp v2.0” and the “QX DNA Alignment Marker 15bp‐5kb” alignment marker (Qiagen). Concentrations were measures with the Qubit, as above for the extraction products. Samples were therefore mixed equimolarly and sequenced on a lane of a NovaSeq 6000 (Illumina, San Diego, California, USA) using a SP300 (2 × 150bp) flow cell. Sequencing was carried out at the NGS Integrative Genomics (NIG) Core Unit of the University of Göttingen.

### 2.3 Nanopore Sequencing and the Assembly of Draft References

Since no plastome of *Armeria* is publicly available yet, we used a freshly collected sample of *A. maritima* (Mill.) Willd. (herbarium voucher GOET065227) to produce Nanopore long reads (ONT; Oxford Nanopore Technologies, Oxford, United Kingdom) and try to assemble draft references for the chloroplast genome and ribosomal DNA (nrDNA). Library preparation was conducted using an ONT Ligation Sequencing Kit SQK‐LSK110 optimized for high throughput and long reads (ONT, Oxford, UK) and applicable for singleplex gDNA sequencing. Initial DNA concentration was adjusted to 1000 ng in 47 μL (ca. 21.5 ng/μL). We followed the manufacturer's instructions for library preparation (protocol v. GDE_9141_v112_revH_01Dec2021, accessible via community.nanoporetech.com) with the few modifications applied as described in Karbstein et al. (2023). Accordingly, incubation times were increased up to 15 min, ethanol wash buffer concentration was increased to 80%, and we enriched DNA fragments of more than 3 kilobase (kb).

We used a MinION 101B device and the ONT software MiniKnow v. 21.11.9 installed on a local Linux system. We loaded the library into a reused R9.4.1 flow cell following the manufacturer's instructions for priming and loading. Prior to loading the library, a hardware check was performed to make sure that the quality of the flow cell was acceptable. Sequencing was run for 72 h. Base calling was done on the local HPC cluster of the University of Göttingen. (GWDG, Göttingen, Germany), using the ONT software GUPPY v. 6.0.1 and the configuration file “dna_r9.4.1_450bps_hac.cfg” (i.e., “high accuracy” base calling).

We performed plastome assembly with the pipeline ptGAUL (Zhou et al. 2023) available at: https://github.com/Bean061/ptgaul), using the plastome of *Limonium bicolor* (Bunge) Kuntze (MZ147631.1) as reference and the default settings, apart from the coverage (−c), which was set to 60. As for the ribosomal DNA (nrDNA), we used the following approach. The fastq file was submitted to PORECHOP v. 0.2.4 for adapter trimming (available at https://github.com/rrwick/Porechop), with the “discard_middle” option turned on. Trimmed reads were then subjected to length and quality filtering using CHOPPER v. 0.2.0 (De Coster and Rademakers 2023), discarding all reads shorter than 500 bp (‐‐minlength 500) and with average reads quality lower than 7 (−q 7). Finally, reads were assembled with CANU v. 2.2 (Koren et al. 2017), with genome size set to 155 kilobases (kb), and “minOverlapLength” set to 300 bp. Assembled contigs were blasted against the nrDNA sequence of *Psylliostachys suworowii* (Regel) Roshkova (AJ132446.1; (Fuertes Aguilar et al. 1999) using BLASTN v. 2.5.0 (Zhang et al. 2000). A single contig of length equal to 15,648 bp matched the reference nrDNA sequence. Both draft references are available at the Gottingen Research Online (GRO.data) repository (doi: 10.25625/LPMQEZ).

### 2.4 Assembly of Illumina Reads

Adapters and low‐quality reads were removed using Trimmomatic v. 0.33 (Bolger et al. 2014). Duplicate reads were eliminated using FastUniq v. 1.1 (Xu et al. 2012). The resulting quality‐trimmed reads were submitted to different mapping strategies. Primarily, we tried to assemble plastomes and nuclear ribosomal DNA de novo, using the software program GetOrganelle v. 1.7.7 (Jin et al. 2020). We used the “embplant_pt” and “embplant_nr” databases, respectively, and k‐mer sizes ranging from 21 to 115. The maximum number of extension rounds was set to 30, and the maximum number of reads (‐‐max‐reads) was increased to 7.5e^7^.

Since in all cases this approach failed reconstructing complete chloroplast genomes and nrDNA sequences, we proceeded mapping Illumina reads on the draft plastome and nrDNA references obtained from Nanopore reads. For instance, draft references obtained from long Nanopore reads can be extremely useful for reference‐based mapping of Illumina short reads when the amount of Illumina data is too low for de novo approaches to reconstruct complete plastomes (Tomasello et al. 2024). Illumina reads were therefore mapped using BWA v. 0.7.16a (Li and Durbin 2009) with default options. Consensus sequences were produced with ConsensusFixer v. 0.4 (available at: https://github.com/cbg‐ethz/consensusfixer), with the “minimum relative abundance of the alternative base to call a wobble” set to 0.3, the “minimal coverage to call consensus” to 5, and the “minimal coverage to call insertion” to 20.

Since cpDNA is maternally inherited in *Armeria* (Nieto Feliner et al. 2002) and nuclear multicopy nrDNA is well known for issues of homogenization caused by concerted evolution (Liao 1999), we explored further the sequence data to find nuclear single‐copy loci. For this purpose, we used CAPTUS (Ortiz et al. 2023), a tool that employs both de novo and reference‐based assembly for the assembly of phylogenomic datasets based on Illumina sequencing data. To clean the raw reads from low‐quality reads and adapters, we used the “captus_assembly clean” step with standard settings. Subsequently, the “captus_assembly assemble” command was employed to assemble the resulting quality‐trimmed reads using MEGAHIT v.1.2.9 (Li et al. 2015). To check if any of the loci of the Angiosperm353 set (Johnson et al. 2018) were present in all samples, contigs from those target regions were extracted using the “captus_assembly extract” function and the “‐nuc_refs” flag. To look for any other single‐copy nuclear region, the “‐‐cluster_leftovers” flag was activated, setting a minimum of five samples (“‐‐cl_min_samples” = 5) and a number of copies per sample equal to one (“‐‐cl_max_copies” = 1). Therefore, we aligned the extracted loci using the “captus_assembly align” command with standard settings. However, none of the loci of the Angiosperm353 were found, probably due to the low depth of our sequencing. Instead, 42 other nuclear single‐copy nuclear regions were found, aligned, and checked with a custom script. Only those that presented a single orthologous sequence for all the seven samples (36 in total) were used in the phylogenetic analyses.

### 2.5 Phylogenetic Analyses

All assembled plastomes, nrDNA, and sequences of the 36 single‐copy nuclear regions were placed in different FASTA files, respectively, which were then aligned using MAFFT v. 7.305b (Katoh and Standley 2013). Alignments were visually checked in AliView v. 1.20 (Larsson 2014), and cases of misaligned positions in guppy regions were eventually corrected. A few cases of sequence inversions were detected in the chloroplast genome alignment (cpDNA) and manually corrected. As already pointed out in previous studies, small inversions in cpDNA alignments are highly homoplastic (Kelchner and Wendel 1996) and can lead to spurious phylogenetic results (Escobari et al. 2021).

We inferred maximum likelihood (ML) phylogenetic trees in RAXML‐NG v. 1.2.0 (Kozlov et al. 2019). For the cpDNA alignment, which was treated as a single partition consisting of 157,577 bp, we used the GTR + G sequence evolution model and 1000 bootstrap (bs) replicates. We included the plastome of *Limonium bicolor* (MZ147631.1) as an outgroup. The orientation of the small single‐copy region (SSC) was here inverted in order to make it match with the *Armeria* sequences.

The nrDNA alignment consisted of 12,414 bp, spanning from one external transcribed spacer (ETS) to the other, and including the 18S ribosomal region, the internal transcribed spacer 1 (ITS1), the 5.8S ribosomal region, ITS2, and the 26S ribosomal region. These regions were treated as different partitions, and the best‐fitting evolution model for each partition was inferred in MODELTEST‐NG v. 1.1.0 (Darriba et al. 2020). Therefore, the ML analyses were run with 1000 bootstrap replicates. The nrDNA sequence of *Psylliostachys suworowii* (AJ132446.1; consisting of the only ITS1, 5.8S and ITS2) was included and used as an outgroup.

The 36 single‐copy nuclear genes produced an alignment that was, on average, 438 base pairs long (171–874 bp; see Table S1). Maximum Likelihood gene trees were inferred for each locus separately with RAXML‐NG, using the GTR + G sequence evolution model and 1000 bootstrap replicates. Those gene trees, and the bootstrap tree produced during the analyses, were used as input for the Coalescent‐based species tree/network analyses (see paragraph below). A total‐evidence analysis was also run, concatenating all the 36 nuclear loci and the nrDNA, using (as above) RAXML‐NG, the GTR + G sequence evolution model, and 1000 bootstrap replicates.

To examine the nuclear phylogenetic trees for conflicting or poorly informative branches, and to pinpoint branches of interest by gene flow, we used the quartet sampling (QS) method (Pease et al. 2018) applying 100 replicates per branch. Quartet sampling results in branch‐specific values that indicate: (i) how many QS replicates produced a quartet topology that is concordant with the input phylogeny (Quartet Concordance [QC]); (ii) estimate if the frequencies of the two possible discordant topologies are equal or skewed toward one discordant topology (Quartet Differential [QD]; i.e., skewed results indicating gene flow as cause of the incongruence); (iii) and specify the percentage of informative QS replicates (Quartet Informativeness [QI]) (Pease et al. 2018).

### 2.6 Coalescent‐Based Species Tree and Likelihood Calculations

To better deal with gene tree incongruences caused by stochastic processes intrinsic to evolution (e.g., Incomplete Lineage Sorting [ILS], concerted evolution), we inferred a coalescent‐based species tree with the software program ASTRAL v. 5.7.8 (Zhang et al. 2018). As input, we used 100 bootstrap trees from the nrDNA analyses and the 36 single‐copy nuclear genes (3700 trees in total). The analyses were run with the “‐t 2” flag on, that is, to calculate also quartet support values for all branches and thus get some hints of the possible causes of gene tree conflict.

Since results of the phylogenetic analyses from different genomic regions may have suggested the presence of gene flow and especially hybrid origin of samples ST06 (*A. saviana*) and MF03 ( *A. denticulata* ), we calculated gene tree probabilities for the species tree scenario (no presence of hybridization) and the phylogenetic networks having the two above‐mentioned samples as hybrids. We used the ASTRAL species tree as null hypothesis (no hybridization). The networks having either ST06, MF03, or both as hybrids were derived from the ASTRAL tree by adding the second parental hybridization branch following the results of the different phylogenetic analyses. Accordingly, the parental contribution of *A. gracilis* (SU04 in the analyses) was added for ST06, and the contribution of *A. arenaria* subsp. *praecox* (MP06) for MF03 (Figure S1). Gene tree probabilities (and therefore species tree/network likelihoods) were calculated in PHYLONET v. 3.8.2 (Than et al. 2008; Wen et al. 2018), using the command “CalGTProb” (Yu et al. 2012), and using as input 100 bootstrap trees from the nrDNA and the 36 single‐copy nuclear genes. As in Karbstein et al. (2022), likelihood scores of the different scenarios were then compared using the Akaike information criterion (AIC). Accordingly, the number of parameters of the different scenarios consisted of the number of branch lengths estimated in the ASTRAL tree plus the number of reticulations of the networks (i.e., one parental contribution pro reticulation estimated in PHYLONET). Models receiving lower AIC scores fit the data better and a difference higher than 2 was considered a strong evidence for preferring the more parameter‐rich model (Burnham and Anderson 2002; Symonds and Moussalli 2011).

### 2.7 Morphometric Analysis

A total of 29 quantitative morphological characters (Table 2) was analyzed, yielding a dataset comprising 134 individuals and 29 variables. Macroscopic measurements were taken using a digital caliper (with an error margin of ±0.1 mm) under a Leica A60 stereomicroscope. Microscopic measurements were obtained from bar‐scaled images in Fiji 2.1.0 (Schindelin et al. 2012). To objectively count the number of leaf veins, free‐hand transverse sections of leaves were made. A “vein” was defined as each fascicle composed of xylem and phloem surrounded by sclerenchyma. The anatomy of summer leaves was examined under a Leitz Diaplan light microscope at 40× magnification. Data were analyzed using R version 4.3.2 and the RStudio IDE (Posit team 2022). We used the *tidyverse* meta‐package (Wickham et al. 2019) for data exploration, preparation, and description. Strong population‐wise outliers greater than 4 Inter Quartile Range (IQR) were double checked using “create_outlier_datatable”, a custom‐made function for data preparation. After that, we used two different approaches. In the first approach, we used *mclust* (Scrucca et al. 2016) for fitting a Gaussian Mixture model giving the seven populations as grouping units. Using the Monte Carlo method, we simulated 1.000.000 herbarium specimens from each estimated probability density (i.e., the populations) to compute the population‐wise multivariate morphometric Jensen–Shannon distance (JSDist) matrix (see Tiburtini, Scrucca, et al. 2024 for details). Then, we used such distance matrix to compute a Neighbor‐Net of morphological data that, unlike traditional dendrograms trees—which assume a strictly bifurcating tree structure—can represent more complex relationships by allowing splits to overlap and form networks. This has been done using the NeighborNet function in the *phangorn* R package (Schliep 2011). In the second approach, we performed a standard linear discriminant analysis fitted with the MASS R package (Venables and Ripley 2002) and plotted with ggplot2.

**TABLE 2:** List and description of the morphometric characters measured in the studied *Armeria* populations.

Character	Description	Tool
AWN_LENG	Awn length (mm)	Fiji
DIAM_CAP	Diameter of the capitulum (mm)	Caliper
LENG_CAL_PED	Length of the calyx pedicel (mm)	Fiji
LENG_CAL_TUBE	Length of the calyx tube (mm)	Fiji
LENG_INNER_SCAL	Length of the inner scale of the involucrum (mm)	Caliper
LENG_INNER_SPI_BRACLE	Length of the bracteole of an inner spikelet (mm)	Caliper
LENG_INNER_SPI_BRACT	Length of the bract of an inner spikelet (mm)	Caliper
LENG_INTER_SCAL	Length of the intermediate scale of the involucrum (mm)	Caliper
LENG_OUT_SCAL	Length of the outer scale of the involucrum (mm)	Caliper
LENG_OUTER_SPI_BRACLE	Length of the bracteole of an outer spikelet (mm)	Caliper
LENG_OUTER_SPI_BRACT	Length of the bract of an outer spikelet (mm)	Caliper
LENG_SUM_LEAF	Length of the summer leaf (mm)	Ruler
LIMB_LENG	Length of the limb (mm)	Fiji
N_SCALES	Number of scales that form the involucrum	
N_SUM_VEINS	Numbers of summer leaf veins with sclerenchyma in cross‐section	Microscope
SCA_DIAM	Diameter of the scape at 1 cm from the base (mm)	Caliper
SCA_LENG	Length of the scape (mm)	Ruler
SCAP_NUM	Number of scapes	
SHEATH_LENG	Length of the sheath (mm)	Caliper
WIDTH_CAL_TUBE	Width below the limb of the calyx tube (mm)	Fiji
WIDTH_IAL_SUM	Width of the summer leaf hyaline margin (mm)	Microscope
WIDTH_INNER_SCAL	Width of the inner scale of the involucrum (mm)	Caliper
WIDTH_INNER_SPI_ BRACT	Width of the bract of an inner spikelet (mm)	Caliper
WIDTH_INNER_SPI_BRACLE	Width of the bracteole of an inner spikelet (mm)	Caliper
WIDTH_INTER_SCAL	Width of the intermediate scale of the involucrum (mm)	Caliper
WIDTH_OUT_SCAL	Width of the outer scale of the involucrum (mm)	Caliper
WIDTH_OUTER_SPI_ BRACT	Width of the bract of an outer spikelet (mm)	Caliper
WIDTH_OUTER_SPI_BRATLE	Width of the bracteole of an outer spikelet (mm)	Caliper
WIDTH_SUM_LEAF	Width in the middle of the summer leaf (mm)	Caliper

## 3 Results

### 3.1 Sequencing

Nanopore sequencing produced 7.01 Gb of *fast5 files (116 files in total). After base calling, 164,959 reads were generated with a mean read length of 997 kbp. The longest read was 23.529 kbp, and the overall data produced amounted to 164.52 kbp. After length and quality filtering, 126,568 reads were retained, corresponding to about 157.505 kbp of sequence data. ptGAUL assembled a draft plastome of 152,610 bp. Canu assembled 43 contigs, one of which consisted of the nrDNA (length 15,648 bp). The data obtained from the Illumina sequencing amounted to 50.25 Gbp. The average number of raw reads retained per sample was 69,708,912 (ranging from 48,799,554 to 91,515,056; see Table S2). On average, 4.57% of the reads were excluded by quality filtering and 13.65% were removed after duplicate reads removal (Table S2).

### 3.2 Phylogenetic Analyses

**FIGURE 2:**
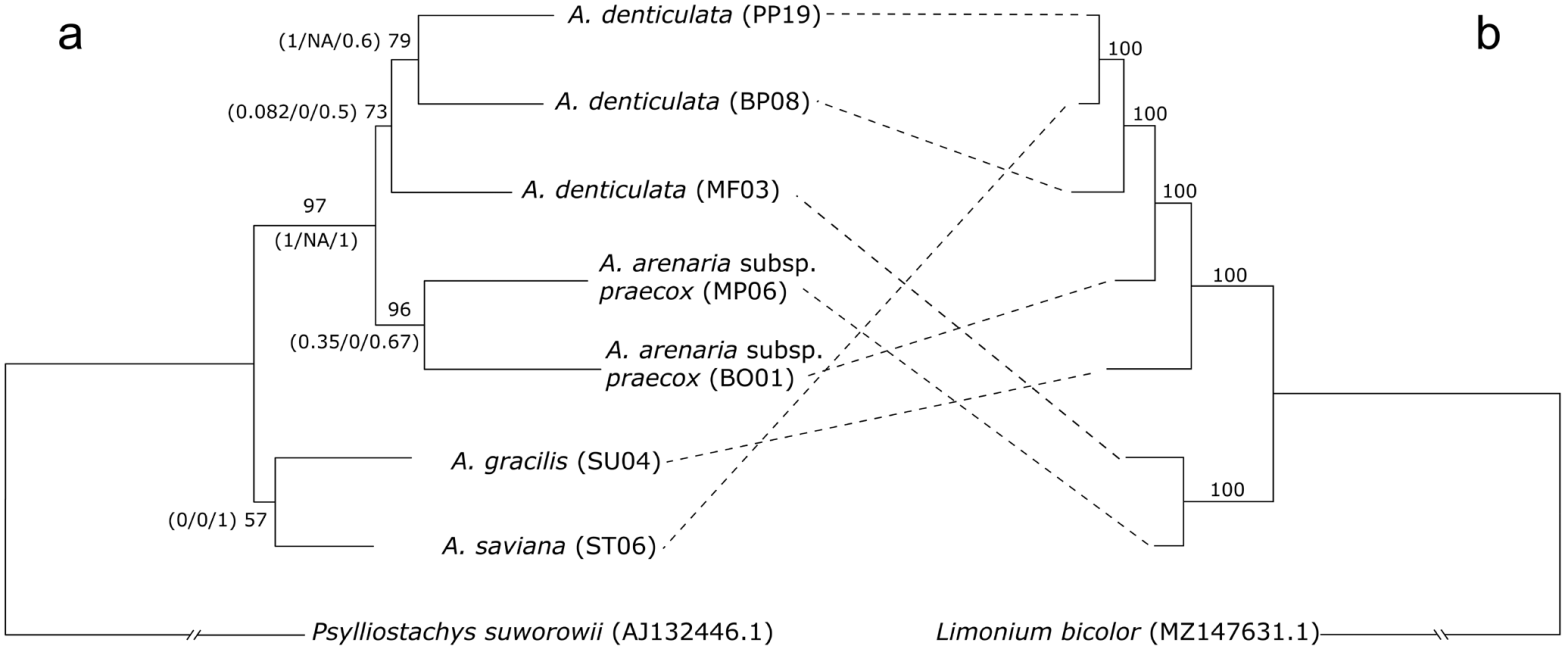
Tanglegram showing (a) the total‐evidence nuclear phylogeny (nrDNA +36 single‐copy nuclear loci) and (b) the cpDNA phylogenetic tree as inferred in RAxML under the Maximum likelihood. Accession codes are explained in Table 1. Numbers above branches indicate bootstrap support values (bs). In the nuclear phylogenetic tree (a), numbers below branches are for the results of the Quartet Sampling analyses and indicate, in order, Quartet Concordance (QC)/Quartet Differential (QD)/Quartet Informativeness (QI). The lengths of the branches leading to the outgroups are shortened. The single original trees are available as (Figures S2–S4).

The ML tree inferred from the cpDNA was fully resolved, with all bs values equal to 100 (Figure 2b). The *Armeria* samples included in the analyses are split into two main clades, one consisting of a sample of *A. denticulata* (MF03) and *A. arenaria* subsp. *praecox* (MP06), and the other including all the remaining samples. The latter clade shows a pectinate topology, with *A. gracilis* (SU05) as an early branch and *A. saviana* (ST06) nested within the remaining samples of *A. denticulata* (BP08 and PP19).

The total‐evidence nuclear phylogeny was to some extent incongruent with the one based on cpDNA, and the reconstructed relationships showed lower support (Figure 2a). An early diverging clade includes *A. saviana* and *A. gracilis* (bs: 57). The remaining samples are subdivided into two clades, one including the accessions of *A. arenaria* subsp. *praecox* (bs: 96) and another with accessions of *A. denticulata* (bs: 73). It is worth noting that the QC and QD values of the branches leading to the *A. saviana*– *A. gracilis* clade, to *A. arenaria* subsp. *praecox*, and to the split between MF03 and the remaining accessions of *A. denticulata* are very low, suggesting that hybridization could be the reason for the low support.

The topology of the nrDNA ML tree was less resolved (Figure S3). However, the clade consisting of *A. gracilis* (SU05) and *A. saviana* (ST06) received here higher support compared with the total‐evidence nuclear phylogeny (bs: 88). In the second clade, consisting of samples of *A. arenaria* subsp. *praecox* (BO01, MP06) and *A. denticulata* (BP08, MF03 and PP19), resolution, as well as bs values, decreases considerably. From the results of the QS analysis, as already observed in the total‐evidence phylogeny, two clades (i.e., a clade consisting of *A. gracilis* SU04 and *A. saviana* ST06, and a clade including *A. denticulata* BP08 and *A. arenaria* subsp. *praecox* BO01) show low QC and QD, but high QI, pointing to gene flow between these two clades. The branch leading to the clade including *A. arenaria* subsp. *praecox* MP06 and *A. denticulata* PP19 received also low QC and QD, but the relatively low QI does not allow making any conclusion on the possible reasons for these incongruences.

### 3.3 Coalescent‐Based Analyses

**FIGURE 3:**
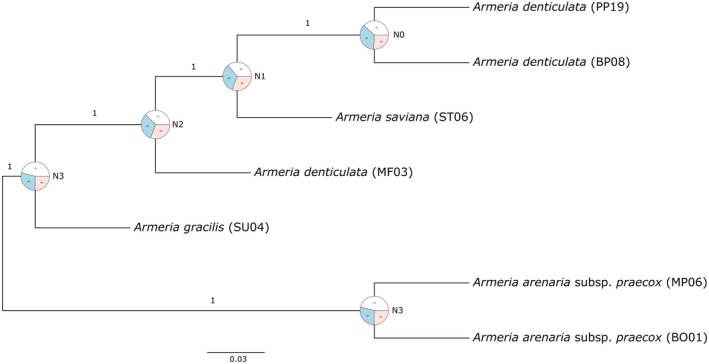
Coalescent‐based species tree as inferred in ASTRAL and based on nrDNA and the 36 nuclear single‐copy regions. Numbers above branches refer to local posterior probabilities. Pie charts at nodes show the quartet scores for the branch leading to the node. White is for the main topology (concordant to the species tree), blue for the first alternative and pink for the second alternative topology. Details on the quartet scores and frequencies as obtained from the ASTRAL analysis are provided in Table S3.

In the species tree obtained in ASTRAL, samples are subdivided into two clades, one consisting of samples of *A. arenaria* subsp. *praecox*, and the other containing the rest of the samples (Figure 3). All branches received a very high local posterior probability; however, quartet scores did not show a prevalence of the main topology (the one shown in the inferred tree) over the two alternative ones (see also Table S3). Although the ASTRAL tree was based only on nuclear data, *A. saviana* is found nested within *A. denticulata* , as in the cpDNA phylogeny. Sample MF3 ( *A. denticulata* ) is found as sister to the clade including *A. saviana* and the rest of *A. denticulata* .

Scenarios treating *A. saviana* and *A. denticulata* (MF03) as hybrids were preferred in the likelihood calculations and AIC scores (Table 3). The scenario in which *A. saviana* is regarded as hybrid received a better AIC score than the tree (58921.67 and 58926.34, respectively). The difference between AIC scores was even higher when treating the sample MF03 as hybrid (network: 58829.13; tree: 58926.34).

**TABLE 3:** Model selection for the scenarios tested in PHYLONET according to likelihood and AIC values. Branches refers to the number of branches for which branch length was interred in ASTRAL, reticulations to the number of hybrid taxa, k to the degree of freedom and AIC to the inferred AIC value.

	Branches	Reticulations	Log‐likelihood	*k*	AIC
Tree (null hypothesis)	5	0	−29458.17	5	58926.34
*A. saviana* hybrid	5	1	−29454.83	6	58921.67
MF03 hybrid	5	1	−29408.57	6	58829.13
Both hybrids	5	2	−29406.94	7	58827.88

### 3.4 Morphometric Analysis

**FIGURE 4:**
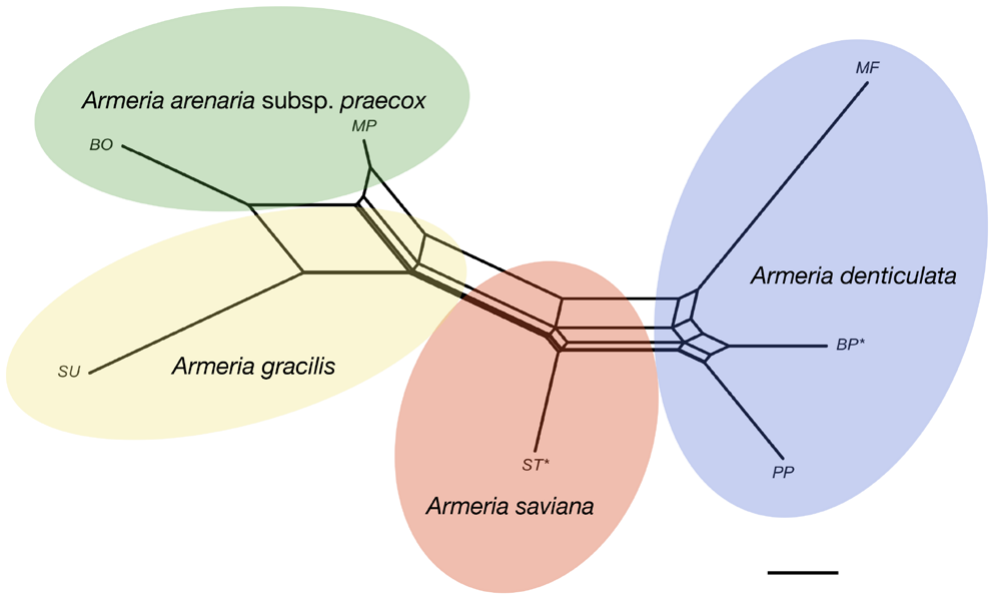
Morphometric Neighbor‐Net calculated from JS distances among populations. Accession codes are explained in Table 1. The bar indicates a 1 p of Euclidean distance. Asterisks indicate type localities.

In Figure 4, the Neighbor‐Net analysis shows four clusters that roughly correspond to the taxa (Table 1). Interestingly, MF is the most differentiated population within *A. denticulata* and occupies a position intermediate between MP ( *A. arenaria* subsp. *praecox*) and the rest of the conspecific populations. The ST population (*A. saviana*) is instead intermediate between SU ( *Armeria gracilis* ) and PP, BP ( *A. denticulata* ). LDA (Figure S5) further corroborates the pattern that emerged with the Neighbor‐Net, and the highest discriminant coefficients for the investigated species are the hyaline leaf margin (WIDTH_IAL_SUM) and limb length (LIMB_LENG) (Figure S6). In particular, *A. arenaria* subsp. *praecox* exhibited relatively short limbs (1.91 ± 0.32 mm) coupled with a wider hyaline leaf margin (0.09 ± 0.02 mm). *Armeria saviana* displayed a comparable limb length (1.92 ± 0.29 mm), but with a slightly narrower hyaline leaf margin (0.07 ± 0.02 mm), while *A. gracilis* showed the longest limbs (2.51 ± 0.30 mm), and comparable hyaline leaf margin (0.07 ± 0.01 mm) (Table S4).

## 4 Discussion

In this study, we aimed to investigate the evolution and speciation patterns within the *Armeria denticulata* complex, with a particular focus on understanding the influence of different substrates on potential hybridization/introgression events. Altogether, the results of the phylogenomic analyses show some degree of incongruence, which might be caused by past hybridization/introgression.

On one hand, the phylogenetic analyses based on the nuclear genome (the total‐evidence tree) (Figure 2a) and the nrDNA phylogenetic analyses (Figure S2) are poorly resolved and show low support values, especially within the *A. arenaria* / *A. denticulata* clade. However, a clear pattern emerges in these analyses, separating *A. gracilis* and *A. saviana* (the two non‐serpentinophyte species) from the rest of the accessions, including all the populations growing on ophiolitic substrates.

On the other hand, the phylogenomic analyses based on the cpDNA, and the coalescent‐based species tree reconstruction (based on nuclear regions) indicate a close relationship between *A. saviana* and *A. denticulata* . In the cpDNA analysis, the haplotype of *A. saviana* is fully nested within those of *A. denticulat* (MF excluded). The fact that *A. saviana* is found as sister to *A. gracilis* in some analyses and nested within *A. denticulata* in others, together with the low QD values received in the Quartet Sampling analyses, could be interpreted as the consequence of a homoploid hybrid origin of this species. Accordingly, *A. saviana* could have originated from a past event of hybridization between a southern population of the serpentinophyte *A. denticulata* (similar to PP in our sampling), that acted as the ovule donor, receiving pollen from some population of the nonserpentinophyte *A. gracilis* . This is further supported by the results of the morphometric analyses, in which *A. saviana* is found closer to *A. denticulata* s.str. but morphologically intermediate between the latter species (more specifically PP) and *A. gracilis* . For example, *A. saviana* exhibits a distinct hyaline margin on the leaves, (0.07 ± 0.02 mm) a trait shared with *A. gracilis* (0.07 ± 0.01 mm) but nearly absent in *A. denticulata* (0.03 ± 0.01 mm). Albeit there is empirical evidence concerning the impossibility for *A. denticulata* to grow outside serpentine outcrops (Selvi 2007), Tauleigne‐Gomes and Lefèbvre (2005) found that hybrids could adapt to environments that can be different from those of the parents, enabling niche expansion (Moore et al. 2021; Pfennig et al. 2016). The hybrid origin of *A. saviana* is further corroborated by the coalescent‐based gene tree probability calculations, which preferred the network scenario seeing *A. saviana* as hybrid over the tree (delta‐AIC < −2; Table 3). Gene tree probabilities calculated under the coalescent model have been demonstrated to be a strong tool for selecting among different topological models (Hime et al. 2020) and/or discriminating between tree‐like and network‐like phylogenies (Wang et al. 2021; Karbstein et al. 2022).

Regarding the relationships between serpentinophyte populations of *A. denticulata* and *A. arenaria* subsp. *praecox*, evidence from different analyses indicates that the two taxa may have gotten in contact in the past. In the total‐evidence nuclear phylogeny and in the nrDNA analyses, samples of the two taxa are found intermingled in the same clade. Unfortunately, *Armeria* is a well‐known case where DNA homogenization due to gene conversion led to the loss of allelic forms from parent taxa in the putative hybrid (Nieto Feliner et al. 2002). As an example, fast homogenization of ITS sequences via concerted evolution was observed in artificial hybrids (F2) between *A. colorata* Pau and *A. villosa* subsp. *longiaristata* (Boiss. & Reut.) Nieto Fel. (Fuertes Aguilar et al. 1999). In the analyses based on the cpDNA, sample MF03 ( *A. denticulata* ) is found in a clade with MP06 ( *A. arenaria* subsp. *praecox*), well separated from the rest of the samples. Thus, in light of the results from different analyses, the MF population of *A. denticulata* from inner Tuscany (Monte Ferrato) could have originated from an introgression/hybridization event between *A. denticulata* s.str. (pollen donor) and a population of *A. arenaria* subsp. *praecox* (ovule donor) similar to MP. In this case, the same ecological adaptation concerning substrate may have facilitated contacts and introgression between the two taxa. Again, our morphometric results highlight that the MF population of *A. denticulata* is morphologically intermediate between the conspecific BP/PP populations and the MP population of *A. arenaria* subsp. *praecox*. The ability of morphometry in capturing hybridization/introgression signals in *Armeria* was previously highlighted in other taxa both in natural populations (Nieto Feliner et al. 2019, 1996; Tauleigne‐Gomes and Lefèbvre 2005) and in common garden experiments (Nieto Feliner 1997). As for the case of *A. saviana*, a hybrid origin of the MF population was strongly supported by the gene tree probabilities calculations (delta‐AIC < −2; Table 3). In addition, several studies to date have shown that stenochorous taxa arose from nearby progenitor taxa (Kay et al. 2011; Otero et al. 2022), and this could be the case for the *Armeria* taxa showing a restricted distribution range in Tuscany.

However, in the present study we have sequenced a single individual per population, which may hamper the possibility of drawing strong conclusions on the hybrid origin and assessing patterns of introgression in the two above‐mentioned populations. Future studies, more focused on one of the two systems, and with a broader, population‐wise phylogenomic sampling, may be needed to corroborate the findings of the present study and assess the extent to which hybridization contributed to the formation of these diverging populations and to the adaptation (in the case of *A. saviana*) to different substrates.

## 5 Conclusions

Our results indicate that the evolutionary processes within the *Armeria denticulata* complex might have been driven by hybridization, which has been here detected for the first time in *Armeria* representatives of the central Mediterranean. More specifically, the nonserpentinophyte *A. saviana* shares the same maternal evolutionary history (cpDNA) with the serpentinophyte *A. denticulata* , but both nrDNA and morphometry suggest a homoploid hybrid origin, involving the surrounding nonserpentinophyte *A. gracilis* , outside the *A. denticulata* complex. In this case, the difference in substrate between the newly generated hybrid and the strict serpentinophyte *A. denticulata* possibly favored isolation and speciation. A single population of *A. denticulata* from inner Tuscany, then, shares the same paternal evolutionary history with other conspecific populations, but cpDNA and morphometry suggest some hybridization/introgression event involving one of the surrounding populations of *A. arenaria* subsp. *praecox* growing on ophiolitic substrates. In this case, the homogeneity of substrates possibly might have favored contact and introgression events, which remained however limited.

Overall, we can conclude that our results support the hypothesis that substrate specificity and hybridization/introgression prompted microevolutionary processes in the *Armeria* taxa endemic to Tuscany.

## Author Contributions

**Manuel Tiburtini:** conceptualization (equal), data curation (equal), formal analysis (equal), methodology (equal), writing – original draft (equal), writing – review and editing (equal). **Salvatore Tomasello:** formal analysis (equal), investigation (equal), methodology (equal), writing – original draft (equal), writing – review and editing (equal). **Eleonora Manzo:** formal analysis (equal), writing – review and editing (equal). **Luca Sandroni:** data curation (equal). **Thomas Abeli:** funding acquisition (equal), writing – review and editing (equal). **Lorenzo Peruzzi:** conceptualization (equal), data curation (equal), funding acquisition (equal), methodology (equal), project administration (equal), supervision (equal), validation (equal), writing – original draft (equal), writing – review and editing (equal).

## Conflicts of Interest

The authors declare no conflicts of interest.

## Supporting information


Appendix S1.

## Data Availability

Morphometric data are available on motivated request. The raw sequencing data are deposited in the European Nucleotide Archive (ENA), with Bio‐Project accession number PRJEB79012. For GenBank accession numbers of the cpDNA and nrDNA sequences of each sample, see Table 1. The cpDNA and nrDNA draft references obtained from Nanopore reads, and the alignments of the 36 single‐copy nuclear genes obtained in CAPTUS, are available at the Göttingen Research Online (GRO.data) repository: https://doi.org/10.25625/LPMQEZ.
