# Supplementary material for: Are Ophiolitic Substrates Drivers for Reticulate Evolution in Armeria (Plumbaginaceae)?
Source: Ecol Evol. 2025 Jun 12;15(6):e71525. doi: 10.1002/ece3.71525 (PMC12162267; doi:10.1002/ece3.71525)
Supplement: Supplementary file 1 — Appendix S1. [file ECE3-15-e71525-s001.docx]

**Are ophiolitic substrates drivers for reticulate evolution in *Armeria* (Plumbaginaceae)?**

Manuel Tiburtini^1^, Salvatore Tomasello^2✻^, Luca Sandroni^1^, Thomas Abeli^3^, Lorenzo Peruzzi^1^

^1^ PLANTSEED Lab, Department of Biology, University of Pisa, Pisa, Italy

^2^ Department of Systematics, Biodiversity and Evolution of Plants (with Herbarium), Albrecht-von-Haller Institute for Plant Sciences, Georg-August-University of Göttingen, Untere Karspüle 2, 37073 Göttingen, Germany

^3^ Department of Earth and Environmental Science, University of Pavia, Via S. Epifanio 14, 00146 Pavia, Italy

Running title: reticulate evolution in *Armeria* serpentinophytes

Keywords: *Armeria,* edaphism, endemism, phylogenomics, reticulate evolution, serpentine, speciation.

^✻^Corresponding Author: [salvatore.tomasello@uni-goettingen.de](mailto:salvatore.tomasello@uni-goettingen.de)

**Table S1.** Information relative to the 36 single-copy nuclear regions found in CAPTUS. Alignments are available at the Göttingen Research Online (GRO.data) repository: https://doi.org/10.25625/LPMQEZ

| **locus** | **Length (bp)** | **invariant sites** |
| --- | --- | --- |
| captus0006 | 437 | 87.19% |
| captus0032 | 484 | 96.9% |
| captus0244 | 250 | 91.6% |
| captus0336 | 341 | 90.91% |
| captus0369 | 437 | 96.34% |
| captus0516 | 217 | 88.94% |
| captus0739 | 296 | 93.92% |
| captus0789 | 197 | 96.95% |
| captus0950 | 348 | 93.97% |
| captus1030 | 320 | 97.81% |
| captus1417 | 368 | 97.83% |
| captus1564 | 387 | 92.25% |
| captus1718 | 344 | 95.64% |
| captus1728 | 302 | 90.73% |
| captus1749 | 459 | 96.3% |
| captus1752 | 760 | 93.16% |
| captus1966 | 349 | 95.99% |
| captus2000 | 793 | 94.2% |
| captus2067 | 171 | 85.96% |
| captus2147 | 459 | 93.9% |
| captus2293 | 702 | 97.72% |
| captus2470 | 658 | 90.58% |
| captus2684 | 504 | 91.07% |
| captus2692 | 287 | 90.24% |
| captus2694 | 436 | 91.51% |
| captus2821 | 547 | 92.69% |
| captus2976 | 551 | 90.93% |
| captus3136 | 442 | 90.72% |
| captus3159 | 427 | 97.66% |
| captus3226 | 381 | 93.96% |
| captus3240 | 673 | 97.03% |
| captus3268 | 365 | 92.6% |
| captus3377 | 536 | 90.49% |
| captus3569 | 864 | 97.34% |
| captus3763 | 322 | 90.99% |
| captus3908 | 344 | 93.6% |

**Table S2**. Reads descriptive statistics of the studied samples including total number of reads and the corresponding Gbp (Giga base pairs), percent of low-quality trimmed reads and duplicates, and the amount of reads passing the quality and duplicate filtering. The length of the plastome (cpDNA) and the ribosomal DNA (rDNA) sequences is also indicated.

| **Sample** | **Nr. reads** | **Gbp** | **% quality trimmed reads** | **% duplicates** | **Survived reads** | **cpDNA length** | **nrDNA length** |
| --- | --- | --- | --- | --- | --- | --- | --- |
| *A. arenaria* subsp. *praecox* BO01 | 91,515,056 | 9.46 | 4.47 | 15.54 | 73,848,738 | 152,539 | 12,407 |
| *A. arenaria* subsp. *praecox* MP06 | 48,936,586 | 3.69 | 7.65 | 13.79 | 38,962,295 | 152,453 | 12,393 |
| *A. denticulata* BP08 | 71,772,234 | 7.66 | 4.03 | 13.17 | 59,809,271 | 152,623 | 12,386 |
| *A. denticulata* MF03 | 77,842,340 | 8.26 | 4.18 | 12.81 | 65,044,068 | 151,976 | 12,407 |
| *A. denticulata* PP19 | 74,973,474 | 7.65 | 3.57 | 13.68 | 62,411,750 | 151,538 | 12,383 |
| *A. gracilis* SU04 | 48,799,554 | 5.17 | 4.25 | 13.53 | 40,406,140 | 152,580 | 12,407 |
| *A. saviana* ST06 | 74,123,142 | 8.36 | 3.85 | 13 | 62,012,858 | 151,967 | 12,408 |

**Table S3**. Results from the ASTRAL quartet scores and frequencies for the tree in Fig. 3. Nodes (N0-N1) refer to those in Fig.3. Scores and frequencies are shown for the main topology (t1) and the two alternative ones (t2 and t3).

| Node | Quartet score | | | Quartet frequency | | |
| --- | --- | --- | --- | --- | --- | --- |
|  | t1 | t2 | t3 | t1 | t2 | t3 |
| N0 | 0.38 | 0.36 | 0.26 | 1405.25 | 1338.75 | 956.00 |
| N1 | 0.36 | 0.33 | 0.31 | 1337.33 | 1228.00 | 1134.67 |
| N2 | 0.37 | 0.32 | 0.31 | 1384.83 | 1182.00 | 1133.17 |
| N3 | 0.46 | 0.29 | 0.25 | 1706.50 | 1056.00 | 937.50 |


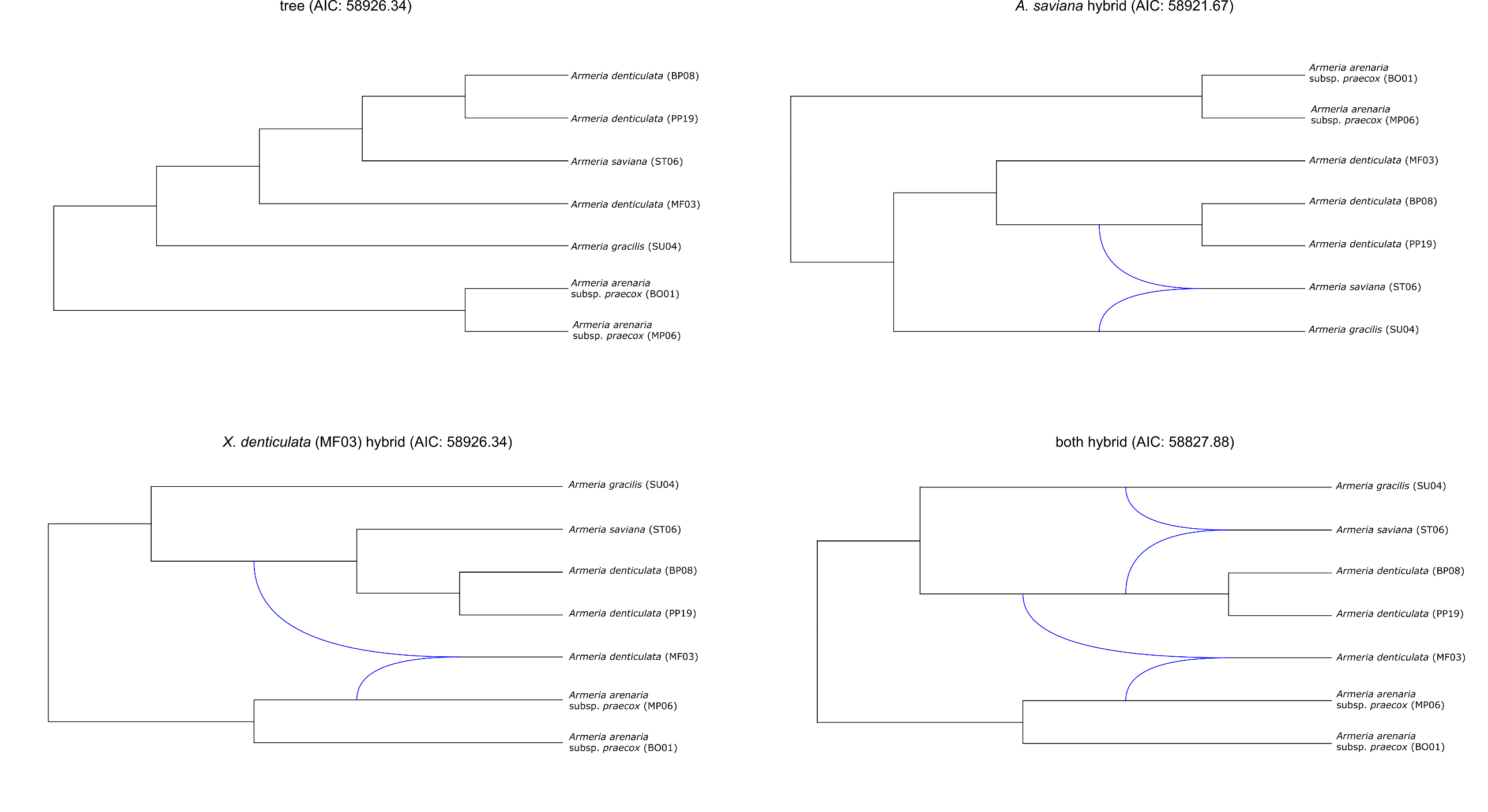
**Fig. S1**. Networks used to calculate gene tree probabilities (and species tree/networks likelihoods) with the command CalcGTProb in Phylonet. The calculations were based on the nrDNA region and the 36 single-copy nuclear loci. One hundred bootstrap trees per locus from the RAxML analyses were used as input.

**Fig. S2**. cpDNA Maximum Likelihood tree as inferred in RAxML based on the whole plastome alignment. Accession codes are following those in in Table 1. Numbers above branches indicate bootstrap support values (bs).


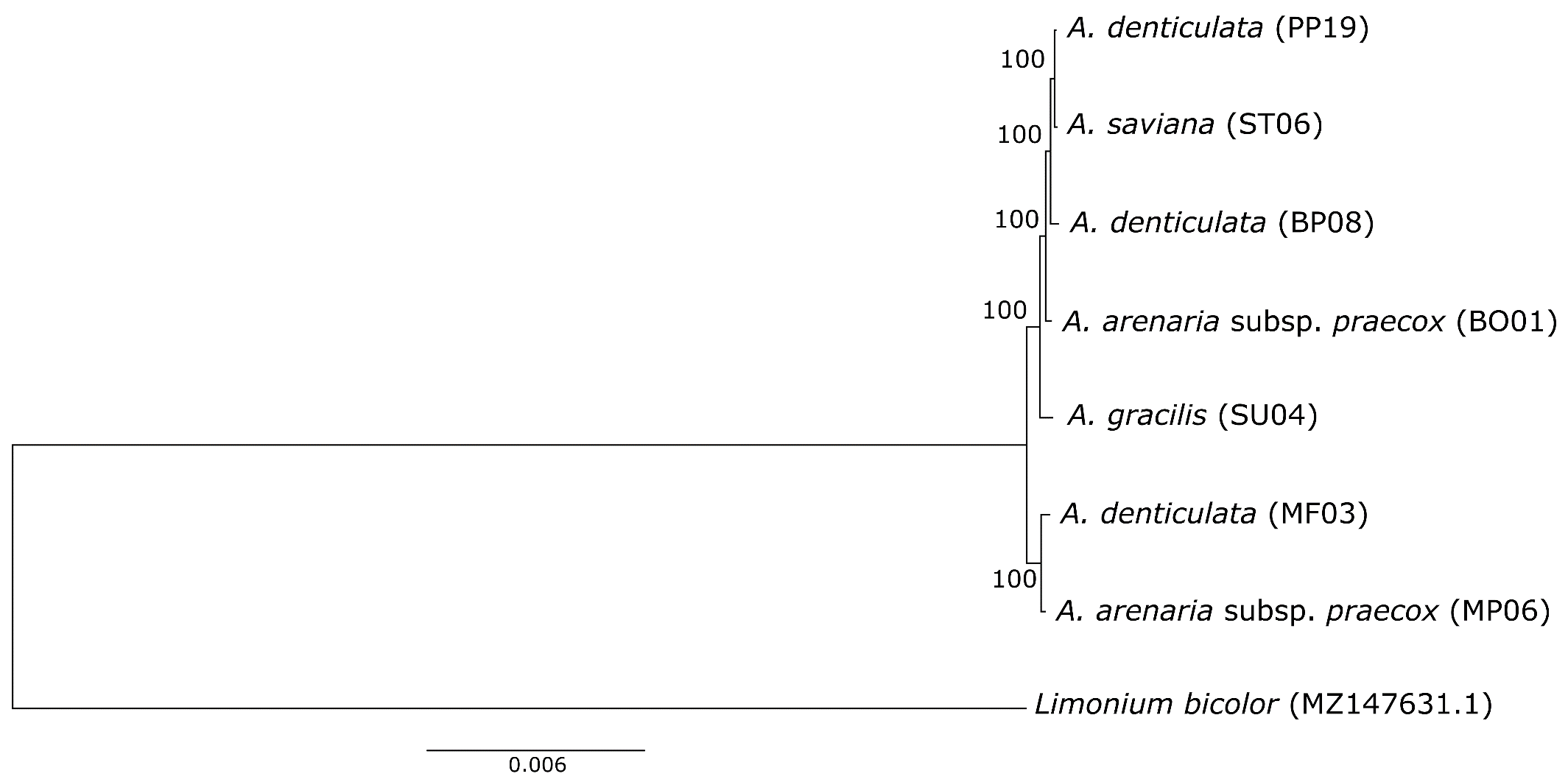


**Fig. S3**. nrDNA Maximum Likelihood tree as inferred in RAxML. Accession codes are following those in in Table 1. Numbers above or beside branches indicate bootstrap support values (bs). Numbers in brackets are for the results of the Quartet sampling analyses, and indicate the Quartet Concordance (QC)/ Quartet Differential (QD)/ Quartet Informativeness (QI) indices, respectively.

**
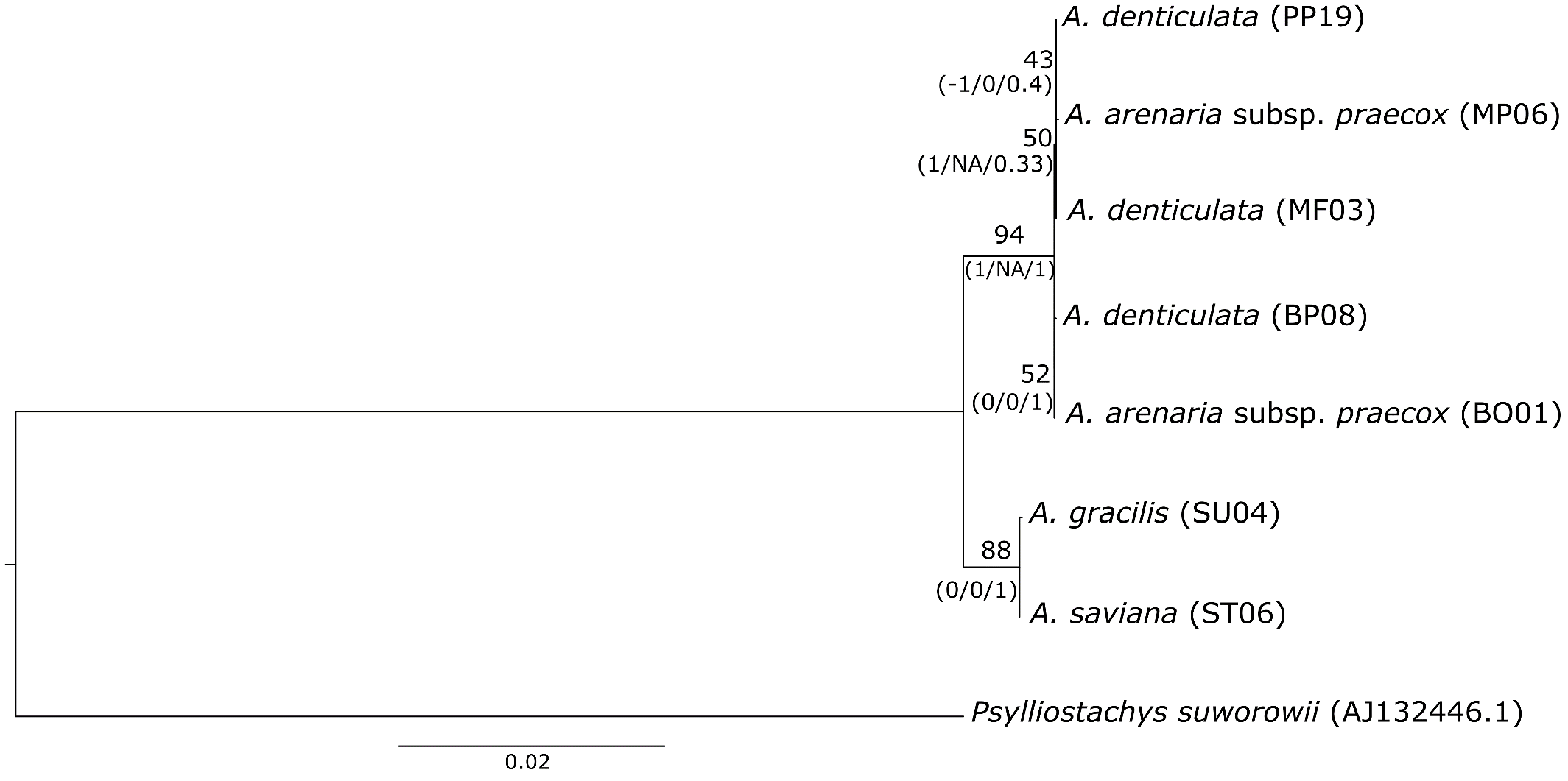
**

**Fig. S4**. RAxML (ML) tree based on the concatenated alignment including the nrDNA region and the 36 single copy nuclear loci. Accession codes are following those in in Table 1. Numbers above or beside branches indicate bootstrap support values (bs). Numbers in brackets are for the results of the Quartet sampling analyses, and indicate the Quartet Concordance (QC)/ Quartet Differential (QD)/ Quartet Informativeness (QI) indices, respectively.


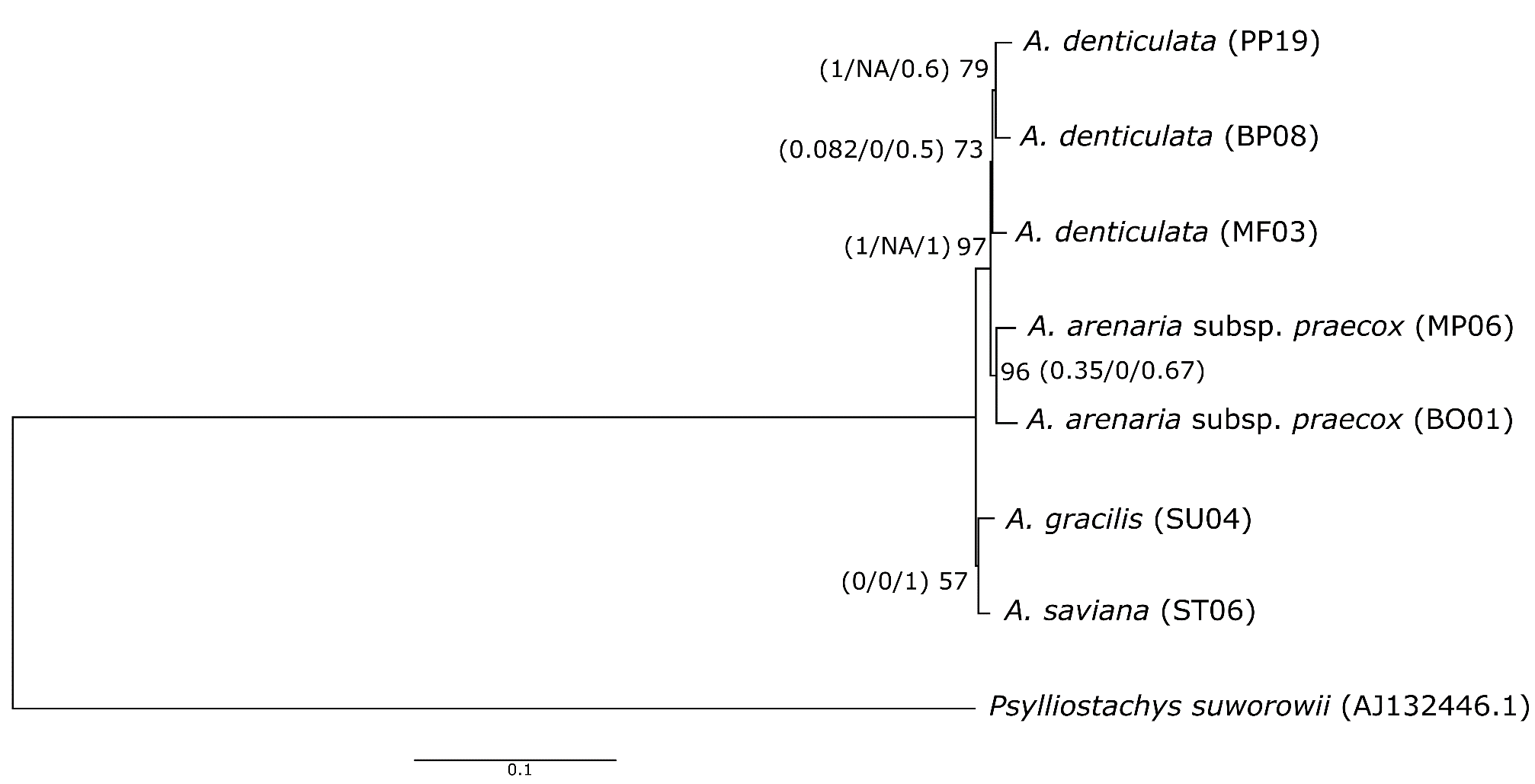


**Fig. S5**. Linear Discriminant analysis (LDA) applied using population as groupings. Colors according to species, shapes according to populations. Asterisks indicate type localities. Green: *Armeria arenaria* subsp. *praecox*; blue: *A. denticulata*; yellow: *A. gracilis*; red: *A. saviana*.


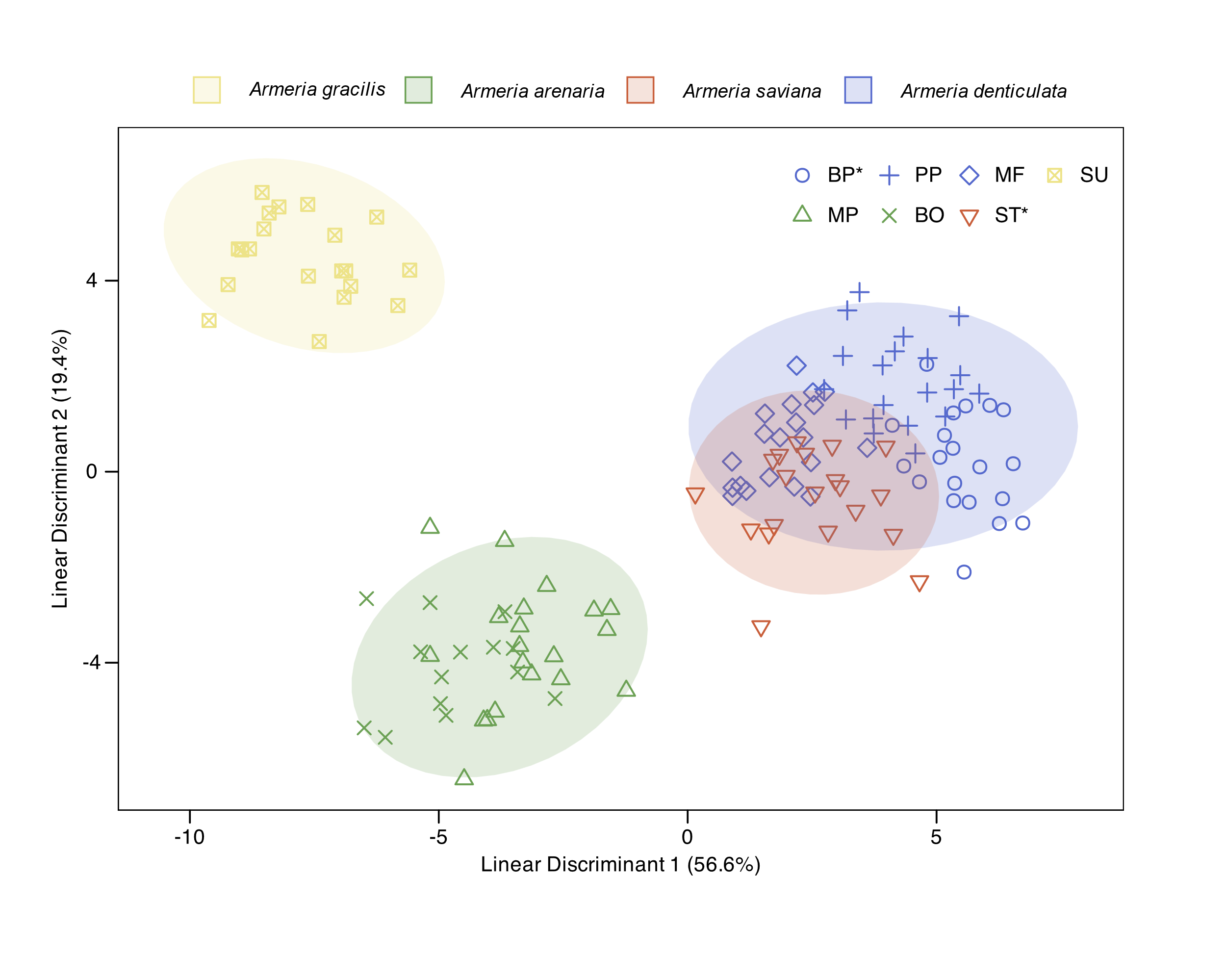


**Fig. S6**. Linear Discriminant coefficients calculated using species as groupings. Discriminant coefficients are normalized using a hyperbolic function (tanh) to force them to range from -1 to 1 and sorted from the most important to the least to improve clarity are readability.

**Table S4** – Mean and standard deviation of the top five most important morphometric variables at both species (A) and population (B) levels according to Figure S6.

| **A) Species** | **WIDTH_IAL_SUM** | **LIMB_LENG** | **WIDTH_CAL_TUBE** | **SCA_DIAM** | **WIDTH_INNER_SCAL** |
| --- | --- | --- | --- | --- | --- |
| *A. arenaria* subsp. *praecox* | 0.09 ± 0.02 | 1.91 ± 0.32 | 1.02 ± 0.14 | 1.37 ± 0.23 | 4.68 ± 0.93 |
| *A. denticulata* | 0.03 ± 0.01 | 1.44 ± 0.25 | 0.74 ± 0.15 | 1.35 ± 0.25 | 2.75 ± 0.65 |
| *A. gracilis* | 0.07 ± 0.01 | 2.51 ± 0.30 | 1.18 ± 0.16 | 1.54 ± 0.23 | 3.17 ± 1.00 |
| *A. saviana* | 0.07 ± 0.02 | 1.92 ± 0.29 | 0.67 ± 0.11 | 1.70 ± 0.36 | 3.35 ± 0.46 |

| **B) Populations** | **WIDTH_IAL_SUM** | **LIMB_LENG** | **WIDTH_CAL_TUBE** | **SCA_DIAM** | **WIDTH_INNER_SCAL** |
| --- | --- | --- | --- | --- | --- |
| BO | 0.09 ± 0.02 | 2.14 ± 0.21 | 1.08 ± 0.14 | 1.50 ± 0.25 | 4.78 ± 0.88 |
| BP* | 0.03 ± 0.01 | 1.49 ± 0.20 | 0.74 ± 0.18 | 1.19 ± 0.22 | 2.71 ± 0.51 |
| MF | 0.03 ± 0.01 | 1.22 ± 0.20 | 0.69 ± 0.14 | 1.41 ± 0.20 | 2.70 ± 0.70 |
| MP | 0.09 ± 0.02 | 1.75 ± 0.28 | 0.97 ± 0.12 | 1.27 ± 0.15 | 4.61 ± 0.98 |
| PP | 0.03 ± 0.01 | 1.61 ± 0.17 | 0.79 ± 0.10 | 1.44 ± 0.27 | 2.83 ± 0.73 |
| ST* | 0.07 ± 0.02 | 1.92 ± 0.29 | 0.67 ± 0.11 | 1.70 ± 0.36 | 3.35 ± 0.46 |
| SU | 0.07 ± 0.01 | 2.51 ± 0.30 | 1.18 ± 0.16 | 1.54 ± 0.23 | 3.17 ± 1.00 |
